# Supplementary material for: Geographic information system protocol for mapping areas targeted for mosquito control in North Carolina
Source: PLoS One. 2023 Mar 24;18(3):e0278253. doi: 10.1371/journal.pone.0278253 (PMC10038244; doi:10.1371/journal.pone.0278253)
Supplement: S3 File — (DOCX) [file pone.0278253.s003.docx]

Source

Address = https://www.nconemap.gov/datasets/nconemap::nc-master-address-dataset-2014

NC Heritage sites = https://www.ncnhp.org/data

Counties = https://www.nconemap.gov/datasets/NCDOT::ncdot-county-boundaries

Census blocks = https://www.census.gov/geographies/mapping-files/time-series/geo/tiger-line-file.html

Rivers = https://www.nconemap.gov/datasets/nconemap::major-hydrography-streams-rivers

Waterbodies = https://www.nconemap.gov/datasets/nconemap::major-hydrography-waterbodies
